# Warning signs of suicide attempts and risk of suicidal recurrence

**DOI:** 10.1192/j.eurpsy.2021.1571

**Published:** 2021-08-13

**Authors:** S. Bader, W. Abbes, M. Tfifha, M. Dhemaid, W. Mahdhaoui, L. Ghanmi

**Affiliations:** 1 Psychiatry, regional hospital of Gabed, gabes, Tunisia; 2 Psychiatry, regional hospital of Gabes, Gabes, Tunisia; 3 Department Of Psychiatry, regional hospital of gabes, gabes, Tunisia; 4 The Department Of Psychiatry, Hospital of gabes, Gabes, Tunisia

## Abstract

**Introduction:**

Detecting warning signs of suicide attempts is a particular difficult task. However, people who plan to commit suicide almost always announce it to someone in some way.

**Objectives:**

Aims of this study were to describe signs preceding the suicide attempt in a group of suicidal persons and its links with suicidal recurrence.

**Methods:**

It was a retrospective study that included all the patients who attempted suicide during the period from May 1st, 2009 to September 25th, 2020 and who were referred to the psychiatry department of the regional hospital of Gabes. Sociodemographic and clinical data as well as suicidal attempts characteristics were assessed.

**Results:**

278 patients were included (female=78.1%), with mean age of 26. The common suicidal attempt method was intentional drug intoxication (67.8%). At least, one clinical manifestation was reported by 75.2% of suicide patients. The most common signs were the tendency to isolation (47.1%), a change in character or behavior (46.6%), thoughts of death (29.6%), anxiety or agitation (24.8%) and recent worsening of the pre-existing psychiatric symptoms (24.3%). Suicidal recurrence affected 24.8% of patients. It was correlated to the presence of a mental disorder (p<10-3), the presence of reflections on death (p=0.02), the onset of a state of anxiety or agitation (p<10-3), recent worsening of pre-existing psychiatric symptoms (p = 0.001) and verbal expression of suicidal thoughts (p<10-3).

**Conclusions:**

The pre-suicidal syndrome is frequently heralded by changes in the patient’s character or behavior. Some suicidal warning signs are associated with the risk of suicidal recurrence.

**Conflict of interest:**

No significant relationships.

